# Supplementary material for: Laughlin anyon complexes with Bose properties
Source: Nat Commun. 2021 Nov 9;12:6477. doi: 10.1038/s41467-021-26873-w (PMC8578557; doi:10.1038/s41467-021-26873-w)
Supplement: Supplementary file 1 — Supplementary information [file 41467_2021_26873_MOESM1_ESM.pdf]

Supplementary Information for

"Laughlin anyon complexes with Bose properties"

L. V. Kulik,[1,2] A. S. Zhuravlev,[1] L. I. Musina,[3,4] E. I. Belozerov,[1,2] A. B. Van'kov,[1,2]

O.V. Volkov,[1] A.A. Zagitova,[1] I. V. Kukushkin,[1] V.Y. Umansky,[5]

[1]*Institute of Solid State Physics Russian Academy of Sciences Chernogolovka, Moscow District, 2 Academician Ossipyan Street, 142432, Russia*

[2]*National Research University Higher School of Economics, Moscow, 20 Myasnitskaya Street, 101000, Russia*

[3]*Moscow Institute of Physics and Technology, 141701, Dolgoprudny, Russia*

[4]*Skolkovo Institute of Science and Technology, Bolshoy Boulevard 30, bld. 1, Moscow, Russia 121205*

[5]*Braun Center for Submicron Research, Weizmann Institute of Science, 234 Herzl Street, POB 26, Rehovot 76100, Israel*

Table of contents

Supplementary Note 1. Numerical spectrum results

Supplementary Note 2. Theoretical model

Supplementary Note 3. Optical transitions in the 18nm quantum well

Supplementary References

Supplementary Note 1. Numerical spectrum results

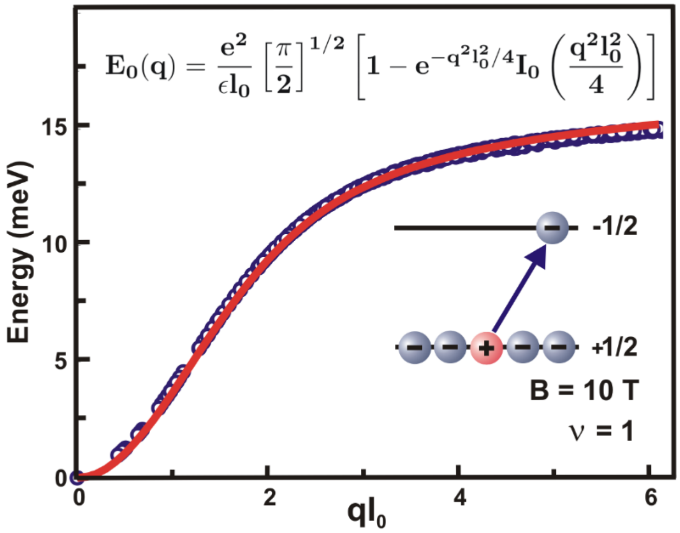

Supplementary Fig. 1| Dispersion dependency of a spin exciton in Hall ferromagnet ν=1. Circles denote the data obtained by Schrodinger's equation for an ideal 2D electron system of 28, 29, or 30 electrons in an external magnetic field of 10 T (single-particle Zeeman energy assumed to be zero, and the geometric form factor is one). The solid line shows the analytical calculation based on the indicated formula from[1], where I_0 is the modified Bessel function, and l_0 is the magnetic length. The inset includes the schematic depiction of a spin exciton.

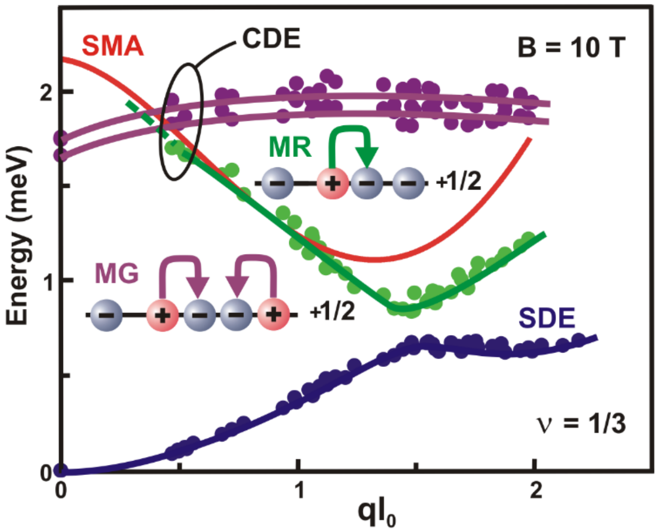

Supplementary Fig. 2| Neutral excitations in the Laughlin state ν=1/3 Energies of the lowest three spin zero excitation branches of magneto-rotons (green dots) and magnetogravitons (purple dots) computed by solving Schrodinger's equation for an ideal 2D electron system consisting of 7, 8, or 9 electrons, in an external magnetic field of 10T. Solid and dashed lines are added for convenience. The solid red curve is the analytical calculation of the magneto-roton dispersion based on the single-mode approximation. Blue dots and the solid

blue line indicate the lowest energy spin one excitation branch. Two insets schematically depict magneto-roton and angular momentum 2 “magneto-graviton”.

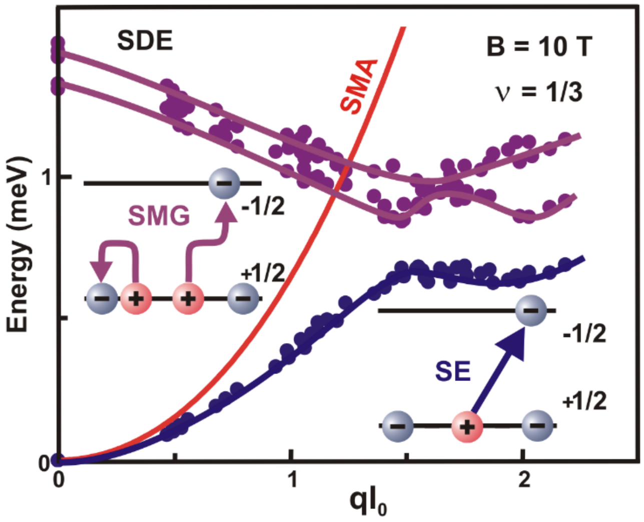

Supplementary Fig. 3| Spin one excitations in the Laughlin state ν=1/3 Energies of the lowest three branches of spin one excitations, spin exciton (blue dots) and spin one “magneto-gravitons” (purple dots), obtained by solving Schrodinger’s equation for an ideal 2D electron system of 7, 8, or 9 electrons, in an external magnetic field of 10 T. The solid red line is the analytical calculation of the spin exciton dispersion based on the single-mode approximation. The insets schematically depict a spin exciton and a spin one “magnetograviton”.

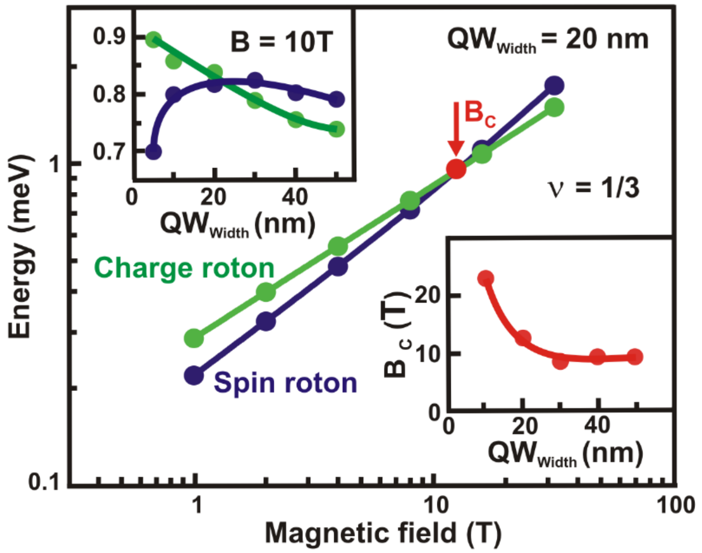

Supplementary Fig. 4| Magnetic field and quantum well width dependencies of roton minima. Energies of roton minima computed for the spin zero and spin one magneto-rotons (green and blue dots, respectively) as a function of the magnetic field by solving Schrodinger’s equation for a 2D electron system of 7, 8, or 9 electrons in the Laughlin state ν=1/3, given the quantum well width of 20 nm. The values of single-particle Zeeman energy for different well widths are taken from ref[2]. The upper inset shows the plot of the same energy for the electron system at 10 T as a function of the quantum well width. In the lower inset, the magnitude of the critical magnetic field, where the energies of the spin zero and spin one roton minima become equal, is plotted versus the quantum well width.

Supplementary Note 2. Theoretical model

In two-dimensional electron systems with relatively weak Coulomb interaction $r_s \lesssim 1$ the ground state of fractional quantum Hall effect with $\nu = 1/3$ is adequately modeled by a partially filled lowest. Landau level. Neutral spin excitations of such system can, nevertheless, combine spin waves and charge excitations within lowest Landau level. Single-particle contribution of such excitations consists only of Zeeman energy, and many-particle one is a combination of exchange and correlation energies, their magnitude is $\sim e^2/\varepsilon\ell_b$. Considering the complex structure of correlations in fractional quantum Hall states, the behaviour of many-particle energy as a function of momentum is troublesome to be described analytically, which is why the method of exact diagonalization of energy spectrum for finite number of electrons was used. For calculating the energies of collective spin excitations, the states on Landau levels with both spin projections were considered.

Many-body states of electrons on Landau levels were considered within torus geometry in a perpendicular quantizing magnetic field[3]. Periodic boundary conditions were chosen as a rectangular elementary cell with dimensions $L_x \times L_y$. It produces a discrete set of wave vectors, satisfying the relations: $k_x = \frac{2\pi}{L_x} \cdot i$ and $k_y = \frac{2\pi}{L_y} \cdot j$, where $i, j \in 0..N_s$. Using dimension ratio $L_x/L_y = 0.95$ allows

to acquire more dots, while this slight asymmetry does not contribute to the physical properties of the system.

In our model the Hamiltonian matrices in Hilbert space of many-body states corresponding to different values of full spin and momentum of the system were constructed to calculate the spectrum of collective excitations with the lowest. energy. Matrix elements were taken in the following form[4]:

$$H_C = \frac{1}{2}\sum_{\alpha,\beta}\sum_{n_1,n_2,n_3,n_4}\sum_{i_1,i_2,i_3,i_4} V^{n_1,n_2,n_3,n_4}_{i_1,i_2,i_3,i_4} \times c^\dagger_{\alpha,n_1,i_1} c^\dagger_{\beta,n_2,i_2} c_{\beta,n_3,i_3} c_{\alpha,n_4,i_4}$$

$$V^{n_1,n_2,n_3,n_4}_{i_1,i_2,i_3,i_4} = \frac{1}{N_s}\frac{e^2}{\epsilon\ell}\sum_{\mathbf{q}\neq 0}\frac{1}{q\ell}\delta'_{i_1,i_4+q_y\ell^2}\delta'_{i_2,i_3-q_y}\ell^2 \times e^{iq_x(i_3-i_1)}F_{n_1,n_4}(\mathbf{q})F_{n_2,n_3}(-\mathbf{q})$$

$$F_{n,n'}(\mathbf{q}) = \frac{\sqrt{min(n,n')!}}{\sqrt{max(n,n')!}} e^{-q^2\ell^2/4} L^{|n-n'|}_{min(n,n')}\left(\frac{q^2\ell^2}{2}\right) \times \left[\frac{\left(\mathrm{sgn}(n-n')q_y + iq_x\right)\ell)}{\sqrt{2}}\right]^{|n-n'|}$$

with $L^k_m(x)$ being associated Laguerre polynomials. The modelling was conducted for the

following parameters of two-dimensional electronic system in GaAs: $\epsilon = 12.5, m^* = 0.067m_0, g = 0.35, n_e = 8 \times 10^{10}$ cm^{-2}. The calculation of matrix elements was made considering the geometric weakening of Coulomb interaction[5]. It was realized via introducing the geometrical form-factor into the two-dimensional Fourier component of Coulomb potential $V(q) = \frac{2\pi e^2}{\varepsilon q} \cdot F(q)$. The function $F(q)$ was calculated using the profile of the envelope wave function of electrons in the lowest dimensional quantization subband of the conduction band. This function was calculated numerically for the actual parameters of the experimental sample and then approximated by the expression: $F(q) = 1.5060801/(q+1) - 0.48825941/(q+1)^2 + -0.02609079$.
It is well known that the calculation of the $\nu = 1/3$ ground state parameters by the exact diagonalization provides an excellent convergence already for electron numbers $N_e = 5,6,7$(ref.[3]). However in this work the calculations are focused on spin-flip excitations at nonzero wave vectors, therefore we had to take into account the states with both spin projections, and also to expand the actual range of momenta in the magnetic Brillouin zone. In order to establish the magneto-roton minimum of SE at momenta $q\ell_B \sim 2$, we had to use a significant number of electrons up to $N_e = 10$ and corresponding Landau level capacity $N_s = 30$. As a result, the shortest dimension of the 1st magnetic Brillouin zone reached values $Q_x\ell_B = \sqrt{\frac{2\pi}{N_x}\frac{N_c}{2}} \approx 2.3$, for sure overlapping the magnetoroton position. Furthermore, the dispersion data are calculated with an excellent detalization, capable to draw the magnetoroton minimum smoothly. The dispersions of excitations for $N_c = 8,9$ are also given on Supplementary figures 2 and 3 for comparison, showing the convergence of the results of numerical simulations.

Supplementary Note 3. Optical transitions in the 18nm quantum well

Following[6] we start from the 4×4 Luttinger Hamiltonian[7] with an external magnetic field B parallel to the z axis, neglecting terms linear in the quasimomentum[8]:

$$\mathbf{H} = \begin{vmatrix} X^+ - 3Z & S^+ & R^+ & 0 \\ S & X^- - Z & 0 & R^+ \\ R & 0 & X^- + Z & -S^+ \\ 0 & R & -S & X^+ + 3Z \end{vmatrix} \quad (1)$$

where:

$$X^\pm = P \pm Q,\ Z = \frac{e}{2c}KB$$
$$P = \frac{\gamma_1}{2}(k_z^2 + k^2),\ Q = \frac{\gamma_2}{2}(-2k_z^2 + k^2)$$
$$R = \frac{\sqrt{3}}{2}\mu k_+^2 - \frac{\sqrt{3}}{2}\bar{\gamma}k_-^2,\ S = \sqrt{3}\gamma_3 k_z k_-$$
$$k^2 = k_x^2 + k_y^2,\ k_1 = k_x \pm ik_y$$
$$\bar{\gamma} = \frac{1}{2}(\gamma_3 + \gamma_2),\ \mu = \frac{1}{2}(\gamma_3 - \gamma_2)$$

In this case, the wave function vector has the following form (the subscripts denote projections of the spin on the z axis):

$$\mathbf{\Phi} = \begin{vmatrix} \phi_{-3/2} \\ \phi_{-1/2} \\ \phi_{+1/2} \\ \phi_{+3/2} \end{vmatrix} \qquad (2)$$

When an external magnetic field B is applied along the z axis direction, the components of the quasimomentum in the plane are no longer commute:
$[k_x, k_y] = -ieB/(\hbar c) = -il^{-2}$, where $l = (\hbar c/eB)^{1/2}$ is the magnetic length. Let us introduce the following ladder operators:

$$a^+ = \frac{l}{\sqrt{2}} k_+,\ a = \frac{l}{\sqrt{2}} k_-,\ N = a^+ a$$

They satisfy relations:

$$[a, a^+] = il^2 [k_x, k_y] = 1$$

The operator N has cigenvalues $0,1,2, \ldots$ The corresponding eigenfunctions are denoted as $\xi_0, \xi_1, \xi_2, \ldots$. These functions satisfy the following relations:

$$a^+ \xi_{n-1} = \sqrt{n} \xi_n,\ a\xi_n = \sqrt{n} \xi_{n-1} \qquad (3)$$

One may rewrite the Hamiltonian (1) in terms of the ladder operators introduced above, neglecting the anisotropic term in R (that contains μ). As was shown in ref.[6], including the anisotropic terms leads to the appearance of anticrossing of Landau levels with numbers that differ by 4. Using the relation $N = \frac{1}{2}(l^2 k^2 - 1)$, one can rewrite the Hamiltonian (1) in the following form:

$$\mathbf{H} = \begin{vmatrix} \Phi_h - 3q & b^+ & -d^+ & 0 \\ b & \Phi_l - q & 0 & -d^+ \\ -d & 0 & \Phi_l + q & -b^+ \\ 0 & -d & -b & \Phi_h + 3q \end{vmatrix} \qquad (4)$$

where we have introduced the notation

$$\Phi_h = \frac{k_z^2}{m_h} + \frac{A}{l^2}\left(N + \frac{1}{2}\right),\ \Phi_l = \frac{k_z^2}{m_l} + \frac{B}{l^2}\left(N + \frac{1}{2}\right)$$
$$q = \frac{K}{2l^2},\ b = \frac{2s}{l} k_z a,\ b^+ = \frac{2s}{l} k_z a^+$$
$$d = \frac{r}{l^2} a^2,\ d^+ = \frac{r}{l^2} a^{+2}$$
$$m_h = \left(\frac{\gamma_1}{2} - \gamma_2\right)^{-1},\ m_l = \left(\frac{\gamma_1}{2} + \gamma_2\right)^{-1}$$
$$A = \gamma_1 + \gamma_2,\ B = \gamma_1 - \gamma_2$$
$$r = \sqrt{3}\bar{\gamma},\ s = \sqrt{\frac{3}{2}}\gamma_3$$

Here, m_h and m_l are doubled effective masses of heavy and light holes. We will seek the solution in the form

$$\Phi = \begin{vmatrix} \xi_n(x,y)\psi_1(z) \\ -i\xi_{n-1}(x,y)\psi_2(z) \\ -\xi_{n-2}(x,y)\psi_3(z) \\ i\xi_{n-3}(x,y)\psi_4(z) \end{vmatrix} \qquad (5)$$

where ξ_n are functions of the coordinates in the (x, y) plane corresponding to the Landau level with number n (eigenfunctions of the operator N), and ψ_i are functions of z that correspond to the various spin projections. Using (3) we rewrite the Hamiltonian (4) in the form of a matrix that acts on the columns of the functions $\psi_i(z)$:

$$\mathbf{H} = \begin{vmatrix} \varepsilon_0 & -\frac{2Q}{l}\frac{\partial}{\partial z} & \frac{N}{l^2} & 0 \\ \frac{2Q}{l}\frac{\partial}{\partial z} & \varepsilon_1 & 0 & \frac{M}{l^2} \\ \frac{N}{l^2} & 0 & \varepsilon_2 & \frac{2I^2}{\partial z} \\ 0 & \frac{M}{l^2} & -\frac{2I^P}{l}\frac{\partial}{\partial z} & \varepsilon_3 \end{vmatrix} \qquad (6)$$

where we have substitute $k_z \to -i\frac{\partial}{\partial z}$ and used the notation

$$M = r\sqrt{(n-1)(n-2)},\ N = r\sqrt{n(n-1)}$$
$$P = s\sqrt{n-2},\ Q = s\sqrt{n}$$
$$E_0 = \frac{A}{l^2}\left(n+\frac{1}{2}\right) - \frac{3}{2}\frac{K}{l^2},\ E_1 = \frac{B}{l^2}\left(n-\frac{1}{2}\right) - \frac{1}{2}\frac{K}{l^2}$$
$$E_2 = \frac{B}{l^2}\left(n-\frac{3}{2}\right) + \frac{1}{2}\frac{K}{l^2},\ E_3 = \frac{A}{l^2}\left(n-\frac{5}{2}\right) + \frac{3}{2}\frac{K}{l^2}$$
$$\varepsilon_0 = -\frac{1}{m_h}\frac{\partial^2}{\partial z^2} + E_0,\ \varepsilon_1 = -\frac{1}{m_l}\frac{\partial^2}{\partial z^2} + E_1$$
$$\varepsilon_2 = -\frac{1}{m_l}\frac{\partial^2}{\partial z^2} + E_2,\ \varepsilon_3 = -\frac{1}{m_h}\frac{\partial^2}{\partial z^2} + E_3$$

This second order differential operator is mapped onto the system of first order differential equations. The easiest method of treating the first derivatives as unknown functions, is somewhat inconvenient here since these functions are not continuous at the boundary between the GaAs quantum well and the AlGaAs barrier. In order to satisfy the requirement of continuity, we construct. new unknown functions by acting on the vector ψ with the velocity operator $v_z = \frac{i}{h}(\mathbf{H}z - z\mathbf{H})$. The continuity of the functions $v_x\psi$ is a consequence of the particles number conservation. Substituting the Hamiltonian of the form

$$\mathbf{H} = A\frac{\partial^2}{\partial z^2} + B\frac{\partial}{\partial z} + C$$

into the velocity operator, one obtains by analogy with ref.[9]:

$$v_z = \frac{i}{\hbar}\left(2A\frac{\partial}{\partial z} + B\right) \qquad (7)$$

Now we can write the complete system of the first-order differential equations for the functions ψ and $\tilde{\psi} = v_z\psi$. The first four equations are obtained directly from (7), while the next four are obtained by differentiating the first four and substituting the expressions obtained for the second derivatives ψ'' into the original Hamiltonian operator (6):

$$\begin{cases} \psi_1' - m_h\left(\tilde{\psi}_1 - \frac{Q}{l}\psi_2\right) = 0 \\ \psi_2' - m_l\left(\tilde{\psi}_2 + \frac{Q}{l}\psi_1\right) = 0 \\ \psi_3' - m_l\left(\tilde{\psi}_3 + \frac{P}{l}\psi_4\right) = 0 \\ \psi_4' - m_h\left(\tilde{\psi}_4 - \frac{P}{l}\psi_3\right) = 0 \\ \tilde{\psi}_1' - \left[\left(A\left(n+\frac{1}{2}\right) - m_lQ^2 - \frac{3}{2}K\right)\frac{1}{l^2} + u - \epsilon\right]\psi_1 + m_l\frac{Q}{l}\tilde{\psi}_2 - \frac{N}{l^2}\psi_3 = 0 \\ \tilde{\psi}_2' - \left[\left(B\left(n-\frac{1}{2}\right) - m_hQ^2 - \frac{1}{2}K\right)\frac{1}{l^2} + u - \epsilon\right]\psi_2 - m_h\frac{Q}{l}\tilde{\psi}_1 - \frac{M}{l^2}\psi_4 = 0 \\ \tilde{\psi}_3' - \left[\left(B\left(n-\frac{3}{2}\right) - m_hP^2 + \frac{1}{2}K\right)\frac{1}{l^2} + u - \epsilon\right]\psi_3 - m_h\frac{l}{l}\tilde{\psi}_4 - \frac{N}{l^2}\psi_1 = 0 \\ \tilde{\psi}_4' - \left[\left(A\left(n-\frac{5}{2}\right) - m_lP^2 + \frac{3}{2}K\right)\frac{1}{l^2} + u - \epsilon\right]\psi_4 + m_l\frac{P}{l}\psi_3 - \frac{M}{l^2}\psi_2 = 0 \end{cases} \qquad (8)$$

where ϵ is the energy eigenvalue and u is the electrostatic potential that should be obtained by solving Poisson equation in z direction for electrons populating the quantum well.

The integral of the wave functions ψ is used as yet another unknown function χ that satisfy equation $\chi' - |\psi|^2$. The last differential equation is introduced for the eigenvalue ϵ. Thus one obtains the remaining two equations:

$$\begin{cases}\chi' - \psi_1^2 - \psi_2^2 - \psi_3^2 - \psi_4^2 = 0 \\ \epsilon' = 0\end{cases} \qquad (9)$$

In order to solve the system of the first order non-linear differential equations like (8,9) numerically we use relaxation method illustrated below. Let us consider the first order differential equation in the generic form:

$$\mathbf{Y}' - \mathbf{F}(\mathbf{Y}) = 0 \qquad (10)$$

where Y is an N -component solution vector defined on the z axis. Let us further pick out M equally spaced points z_k at a distance h from one another within the $\mathrm{Y}(z)$ definition range. Then one can approximate (10) by an equivalent system of finite difference equations for the values of Y at the grid points $\{z_k\}$:
$\mathbf{Y}_k = \mathbf{Y}(z_k)$. These equations are

$$\mathbf{E}_k = \mathbf{Y}_k - \mathbf{Y}_{k-1} - \frac{h}{2}\left(\mathbf{F}(\mathbf{Y}_k) + \mathbf{F}(\mathbf{Y}_{k-1})\right) = 0 \qquad (11)$$

Here E_k and Y_k are $N \times M$ vectors representing N original equations sampled as M grid points.

The general method of solving such non-linear system of equations consists of applying so called Newton iterations starting from some initial approximation based on analytical solution of some trivial case, for. ex. zero magnetic field and quantum well with infinite boundary potential. Let us define an error vector $\Delta_s = \mathbf{E}(\mathbf{Y}_s)$ after the s-th iteration. Then the next solution vector can be written as

$$\mathbf{Y}_{s+1} = \mathbf{Y}_s - \left[\frac{\delta\mathbf{E}}{\delta\mathbf{Y}}\right]^{-1} \Delta_s \qquad (12)$$

The Jacobi matrix $\hat{\mathbf{S}} = \delta\mathbf{E}/\delta\mathbf{Y}$ for this problem is block-diagonal with most. off-diagonal elements equal to zero. We use well known algorithms (see ref.[10]) to invert it efficiently. By recursively applying (12) one can obtain the solution of (10) with desired precision. The transformation from simplified initial approximation to the one having desired physical parameters (for. ex. non-zero magnetic field) should be performed in small steps iteratively solving the equations using previous step results as initial approximation for the next iteration.

Supplementary References

1. Yu. A. Bychkov, S. V. Iordanskii, G. M. Eliashberg, Two-dimensional electrons in a strong magnetic field, *JETP Lett*. 33, 143 (1981).

2. M. J. Snelling et.al., Magnetic g factor of electrons in GaAs/Al_xGa_{1-x}As quantum wells, *Phys. Rev. B* 44, 11345 (1991).

3. F.D.M.Haldane, Phys.Rev.Lett.55, 2095 (1985).

4. Wenchen Luo and Tapash Chakraborty, Missing fractional quantum Hall states in ZnO, Phys. Rev. B 93, 161103(R) (2016) – Published 6 April 2016

5. A. B. Vankov, L. V. Kulik, I. V. Kukushkin, V. E. Kirpichev, S. Dickmann,V. M. Zhilin, J. H. Smet, K. von Klitzing, and W. Wegscheider, Phys. Rev.Lett.97, 246801 (2006).
6. S.-R. Eric Yang, D. A. Broido, and L. J. Sham, Phys. Rev. B 32,6630(1985).
7. J. M. Luttinger, Phys. Rev. 102,1030(1956).
8. D. A. Broido and L. J. Sham, Phys. Rev. B 31,888(1985).
9. R. Eppenga, M. F. H. Schuurmans, and S. Golak, Phys. Rev. B 36,1554 (1987).
10. W. H. Press, S. A. Teukolsky, W. T. Vetterling, and B. P. Flannery, Numerical Recipes in C, Second Edition, Cambridge Univ. Press, 2002.

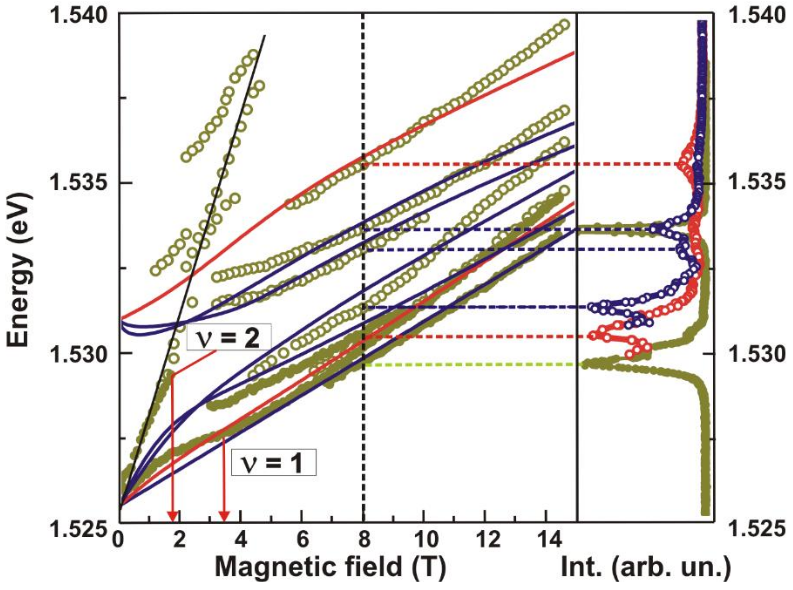

Supplementary Fig. 5| Left, the optical transition energies obtained from photoexcitation spectra (open dots) and photoluminescence (solid dots). The electron density obtained from the optical spectra well agree with the transport data. Blue and rad solid lines show the calculated transition energies in two polarizations of incident photons σ- and σ+, correspondingly. Right, photoexcitation spectra in σ- and σ+ polarizations (open dots) and photoluminescence (solid dots).
